# The “2DE-Pattern” Database for Inventory of Proteoform Profiles: 2026 Upgrade and Update on Outcomes

**DOI:** 10.3390/proteomes14030046

**Published:** 2026-09-07

**Authors:** Stanislav Naryzhny, Nikolay Klopov, Natalia Ronzhina, Elena Zorina, Olga Legina

**Affiliations:** 1 Petersburg Nuclear Physics Institute Named by B. P. Konstantinov of National Research Centre “Kurchatov Institute”, Gatchina 188300, Russia; klopov_nv@pnpi.nrcki.ru (N.K.); ronzhina_nl@pnpi.nrcki.ru (N.R.); olgaleg@mail.ru (O.L.); 2 Institute of Biomedical Chemistry, Pogodinskaya, 10, Moscow 119121, Russia; el.zorina@ibmc.msk.ru

**Keywords:** proteoforms, database, bioinformatics, pattern, 2DE

## Abstract

Background: Modern proteomics faces a critical bottleneck: the vast discrepancy between the number of genes in the human genome and the exponentially greater variety of functional proteoforms that actually drive biological processes. Methods: Our paper addresses the urgent need for high-resolution systematic mapping of these proteoforms, arguing that the true frontier of molecular biology lies in the precise identification and categorization of protein variants. It centers on the development and expansion of the “2DE-pattern” database, a specialized platform designed to bridge the gap between theoretical protein sequences and the physical reality of proteins as captured through two-dimensional electrophoresis (2DE). The “2DE-pattern” database is based on information obtained by separation of proteoforms using 2DE followed by shotgun ESI LC-MS/MS. It was launched in 2020, contains multiple isoform-centric patterns of proteoforms, and can be freely used. Results: Here, we report the additional data and all updates that were added into this database. Also, the database was upgraded to be more research-oriented. Tools were incorporated into the database to allow convenient comparative analysis of the data. Conclusions: New additions and enhancements now allow us to consider our database a knowledge base.

## 1. Introduction

As proteomics delves deeper into the protein heterogeneity associated with a huge number of proteoforms, the amount of information grows very rapidly, so there is high demand for convenient ways to store and use this information [1,2]. The main users of proteomic databases are researchers in the fields of biology, bioinformatics, and clinical science analyzing protein levels, their sequences, and molecular networks. Therefore, databases are a crucial part of proteomics. The most well-known open-access databases are located at the National Center for Biotechnology Information (NCBI; http://www.ncbi.nlm.nih.gov) and on the SIB ExPASy bioinformatics resource portal, https://www.expasy.org/, including, for example, NeXtProt and UniProt (https://www.nextprot.org/, http://www.uniprot.org/) [3,4]. The Human Protein Atlas is another prominent research portal dedicated to mapping all human proteins across cells, tissues, organs, and blood [5]. The Consortium for Top-Down Proteomics (CTDP) is involved in a project aiming to define the complete set of human proteoforms [6,7]. Accordingly, a proteoform database called Proteoform Atlas was organized (http://atlas.topdownproteomics.org) [8,9]. In addition, archive databases such as PRIDE (the PRoteomics IDEntifications database) or PeptideAtlas, where information about proteoforms can be found, are available [10,11]. Typically, the data collected in the databases are based on the specific method by which they were obtained. As for proteoforms, two-dimensional gel electrophoresis (2DE) is ideal for their separation and subsequent identification [12,13,14,15]. Moreover, 2DE allows one to measure the basic parameters of a protein molecule—molecular weight (MW) and pI—and allows for the storage of the obtained information in a highly visually accessible format [16,17,18,19,20]. Although the subsequent analysis is performed using bottom-up mass spectrometry, the overall approach is called top-down integrative proteomics [21,22,23,24,25]. To enable a complete analysis of samples, another approach was developed whereby the entire gel was divided into individual sections, which were then processed and subjected to mass spectrometry. Based on this approach, we generated proteoform profiles for several cell types [26,27,28]. Moreover, these data were used to create a web database called “2DE-pattern” [29]. Although this approach is not as accurate as classical top-down mass spectrometry, it allows one to obtain a general idea of proteoform families, referred to as patterns. Here, we report on more data as well as more features that were incorporated into this database recently. These updates have significantly expanded the database and enhanced its discovery power.

## 2. Materials and Methods

### 2.1. Sample Preparation

Human hepatocellular carcinoma cells (HepG2, collection of Institute of Biomedical Chemistry, Moscow, Russia), embryonic lung fibroblasts (FLEH, Russian Collection of Vertebrate Cell Cultures in Institute of Cytology of the Russian Academy of Sciences, St.-Petersburg, Russia), and glioblastoma (primary line L, generated in the Laboratory of Cell Biology (NRC “Kurchatov Institute”-PNPI, Gatchina, Russia) cells were cultured, collected, and extracted in the same way as was described in detail before [27,30,31,32]. Hepatocellular carcinoma and control tissues (*n* = 5) were extracted according to [28]. The samples contained 2 mg of protein in 100 µL of lysis buffer (7 M urea, 2 M thiourea, 4% CHAPS, 1% dithiothreitol (DTT), 2% ampholytes, pH 3–10, protease inhibitor mixture). The protein concentration in the sample was determined by the method of Bradford [33].

### 2.2. 2DE

All procedures (at least 3 technical replicates) were performed according to the protocols described previously [27,30,31,34]. Briefly, for classical 2DE, 1000 μg of protein was loaded onto a 24 cm long nonlinear IPG strip. After isoelectric focusing (IEF), the separation was produced on a 12% polyacrylamide gel (1 mm × 25 cm × 20 cm) under denaturing conditions. The gel was stained with Coomassie Blue R350, scanned with an ImageScanner III (GE Healthcare, Pittsburgh, PA, USA), and analyzed with an ImageMaster 2D Platinum 7.0 (GE Healthcare).

For sectional 2DE, 500 μg of protein was loaded onto a 7 cm long linear IPG strip. After the second dimension, the gel (1 mm × 8 cm × 8 cm) was stained, scanned, analyzed using ImageMaster 2D Platinum 7.0, and divided into 96 sections with defined coordinates designated as 1–12 along the MW dimension and A–H along the pI dimension. Each section was digested with trypsin. Tryptic peptides were eluted from the gel with extraction solution (5% (*v*/*v*) acetonitrile, 5% (*v*/*v*) formic acid) and dried in a Speed Vac vacuum centrifuge (Thermo Scientific, Waltham, MA, USA). The peptides were dissolved in 5% (*v*/*v*) formic acid.

In the case of semi-virtual 2DE [26,35], the first step was the same as in classical 2DE. After IEF, the strip was cut into 48 equal sections and processed according to the trypsin digestion protocol. Samples were digested overnight at 37 °C. Peptides were extracted with 150 μL of 60% acetonitrile (ACN) and 0.1% trifluoroacetic acid (TFA). The extracts were dried in a Speed Vac vacuum dryer and dissolved in 20 µL of 0.1% TFA before analysis by mass spectrometry (MS). Protein identification and relative quantification were performed using SearchGUI v 4.0.12. A semi-virtual 2D map was constructed according to the theoretical MW and experimental pI of the detected proteins.

The virtual 2DE maps were constructed using data obtained by the so-called Filter-Aided Sample Preparation (FASP) method [28,36]. In this case, the detected proteins were plotted on the map according to their theoretical pI/MW and experimentally detected abundance (emPAI).

### 2.3. ESI LC-MS/MS Analysis

The analysis was completed by the “shotgun” approach as described previously [26,35]. Panoramic proteomic analysis of the obtained extracts was performed using filter processing and subsequent mass spectrometry (FASP). Centrifuge concentrators (Microcon YM–30, Merck, Rahway, NJ, USA) were used for this purpose. In short, extracts containing the required amount of protein (300 μg) were placed in concentrators and sequentially treated with solutions: (a) for the reduction of disulfide bonds (100 mM DTT in 100 mM Tris-HCl, pH 8.5), then (b) for the alkylation of sulfhydryl groups (50 mM iodoacetamide, 8 M urea, 100 mM Tris-HCl, pH 8.5), and (c) for hydrolysis with trypsin (Trypsin Gold, Promega, Madison, WI, USA). Tandem mass-spectrometry analysis was carried out on an Orbitrap Q-Exactive mass spectrometer (Thermo Scientific, USA) according to the protocols described previously, using an Agilent HPLC system 1100 Series (“Agilent Technologies”, Santa Clara, CA, USA) [37].

### 2.4. Protein Identification

Identification of proteins was performed using SearchGUI, an open-source graphical user interface [38]. Two unique peptides per protein were required for all protein identifications. The exponentially modified PAI (emPAI), the exponential form of the protein abundance index (PAI), defined as the number of detected peptides divided by the number of theoretically observable tryptic peptides for each protein, was used to estimate protein abundance [39]. All additional information about the methods can also be reached through the front page of the database by clicking the corresponding links for protocols or articles.

### 2.5. Software Used for Database Construction

All data were stored in a Mysql database (version 15.1) on a Linux server. The scripts that process requests from the user interface were developed using Perl 5 (version 30). The website is based on webserver Apache/2.4.39. The interactive user interface for the database is implemented using HTML, CSS, JavaScript, and JQuery library. The queries to server programs are executed using AJAX technology.

## 3. Results and Discussion

### 3.1. Overview of the Database

**Figure 1:**
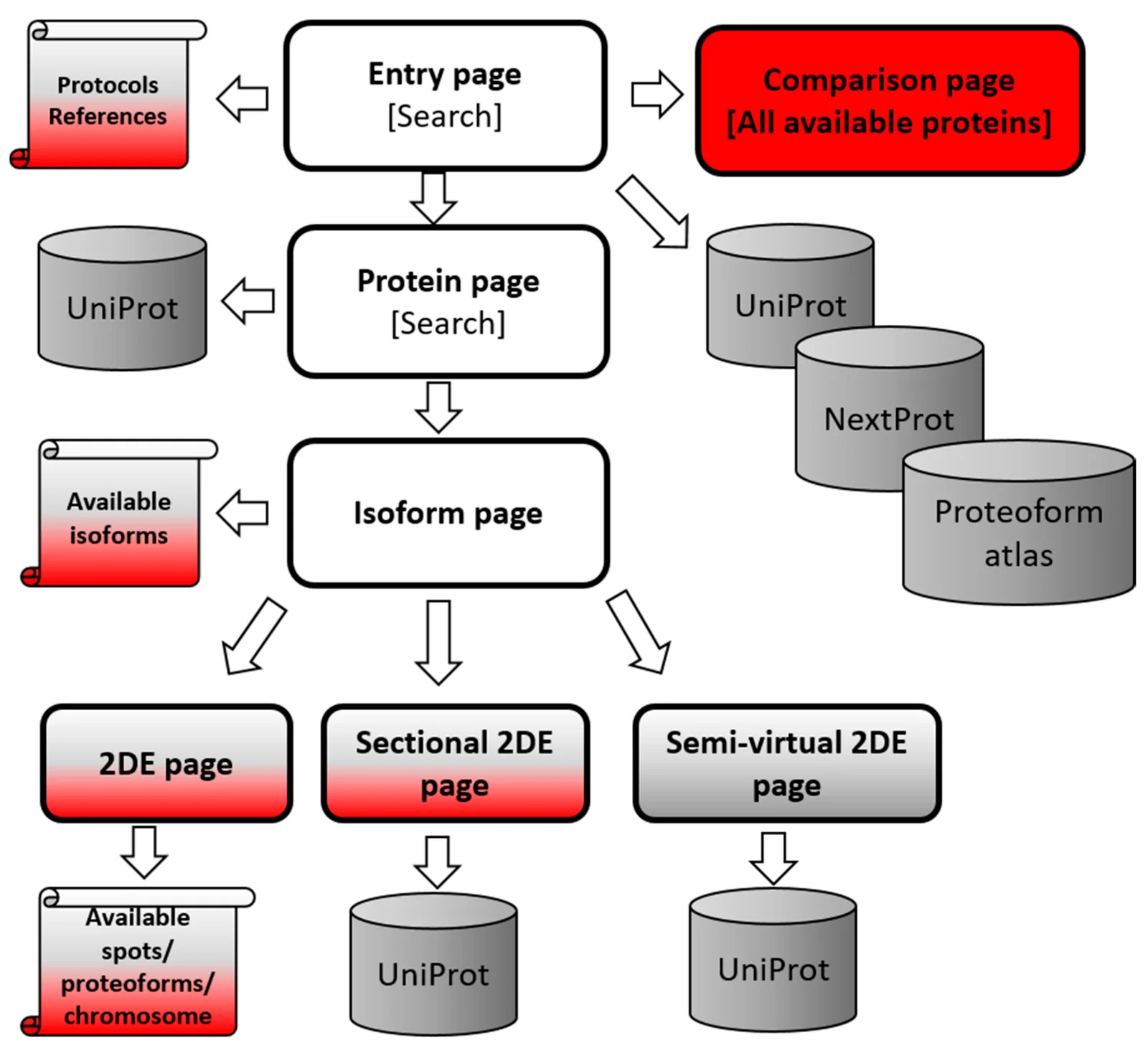
A flow chart of the proteoform database “2DE-pattern”. A user starts from the top, at the “Entry page”, where a choice of search parameters can be found. The next steps can be done by following the arrows. Blocks colored red indicate content that differs from the first edition of the database. The graph is a modified version of that presented in [29].

**Figure 2:**
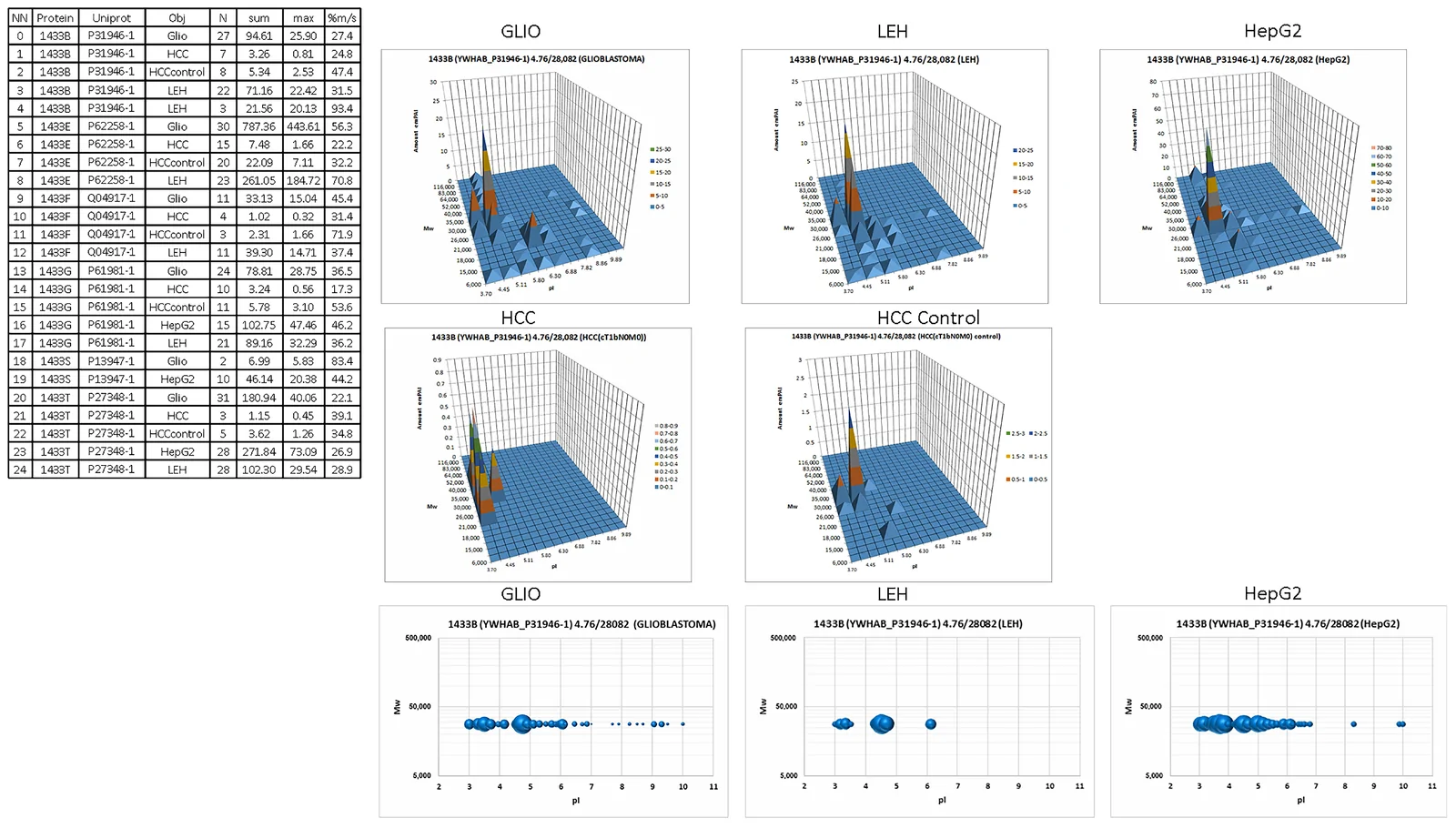
An example of the “Comparison Page” (a partial view). The comparison of the available images can be performed here after selection of the isoform number.

Our database “2DE-pattern” was developed to provide a simple and comprehensive tool to store and analyze information about proteoforms separated according to 2DE principles (Figure 1). These principles are based on the fundamental physicochemical parameters of polypeptides, namely isoelectric point (pI) and molecular weight (MW). Each proteoform (polypeptide) has a specific set of these parameters. Unfortunately, the very useful database SWISS-2DPAGE is not available anymore, and the interactive website https://world-2dpage.expasy.org/swiss-2dpage/ has been discontinued (accessed on 1 January 2026). Accordingly, we are trying to enlarge and upgrade our database. We are using three different approaches based on experimental and virtual 2DE separation and identification using mass spectrometry. The first approach is based on classical 2DE. In addition to the experimental 2DE, we performed virtual 2DE for HCC samples. In this case, the information about protein abundances was obtained by the direct ESI LC-MS/MS analysis of extracts. The virtual 2DE was conducted based on this experimental information and the theoretical parameters (pI, MW) available for each polypeptide. The second one is sectional 2DE, in which a whole gel, not only selected spots, is analyzed by ESI LC-MS/MS section by section. The third one is semi-virtual 2DE, in which proteoforms are separated only by IEF according to their pI. Each approach allows for the production of a specific proteoform pattern for every isoform. All three approaches have limitations, but they are complementary to each other and allow for obtaining a better view of the combined proteoform profiles of isoforms (Figure 2). To generate the complete image of the proteoform landscape, detailed information (sequence and PTMs) about each proteoform should be obtained. But this is a task for the next step in proteoform identification. Currently, the database contains information on proteoform patterns for 8014 isoforms of 7053 proteins. This represents a twofold increase compared with the first version of the database.

### 3.2. Additions to the Database Content and Search Features

Users can search the database using three distinct identifiers from the front (entry) page:

- The UniProt accession number of the target protein.
- The official name of the protein.
- The gene symbol or name.

The home page provides direct links to the underlying data sources:

- Protocols: Detailed laboratory methods used to generate the data.
- References: Published papers validating the database content.

We also added the ability to check the general information about all isoforms included in the database. This can be done by clicking “All available proteins (isoforms)—Click HERE”. This will transfer users to the Comparison Page, where they can evaluate and compare protein expressions:

- emPAI Values: View quantitative data for detected proteoforms.
- Bulk Graph Loading: Render all available charts simultaneously.
- Pattern Comparison: Contrast and compare 2DE maps across different samples.

The data currently originate from five specific sources:

- Glia-L: Glioblastoma cell line.
- LEH: Human embryonic lung fibroblasts.
- HepG2: Hepatocellular carcinoma cell line.
- HCC: Hepatocellular carcinoma tissue samples.
- HCCCONTROL: Healthy control tissue for hepatocellular carcinoma.

The original version of the database contained only Glia-L and LEH.

### 3.3. Navigation Workflow

The database utilizes a hierarchical transition model to navigate from general protein data to specific experimental proteoform patterns:

[Protein Page] ---> [Isoform Page] ---> [Sample Pages (3 Types)]

Protein Page: Displays basic information, SWISS-PROT ID/accession number, description line, and all available isoforms.

Isoform Page: Links to different sample pages and includes a master table mapping isoform data (Figure 1).

Sample Pages: Delivers specialized proteoform patterns based on the chosen analytical approach.

### 3.4. Sample Page Types and Methods

The database offers three types of sample pages determined by the experimental workflow used to analyze the proteome. The first type is classical or virtual 2DE maps:

Classical 2DE Map: This displays physical, experimental gel spots where proteins were detected. In the 2DE map, spots where different proteoforms of the same isoform were detected are highlighted. Accordingly, a proteoform pattern of this isoform is produced. Additionally, basic experimental information about the spot abundance and the isoform (isoform name, protein name, gene name, chromosome, theoretical pI, theoretical Mw, experimental pI, theoretical MW, emPAI, modifications) is shown. Minimal information obtained by mass spectrometric analysis is shown in the table. Also, by clicking on a spot a user can extract information about all the isoforms that were detected in this spot. Additionally, there is an option to check each chromosome for the detected proteoforms. Classical 2DE is used for the samples Glia-L, LEH, and HepG2.

**Figure 3:**
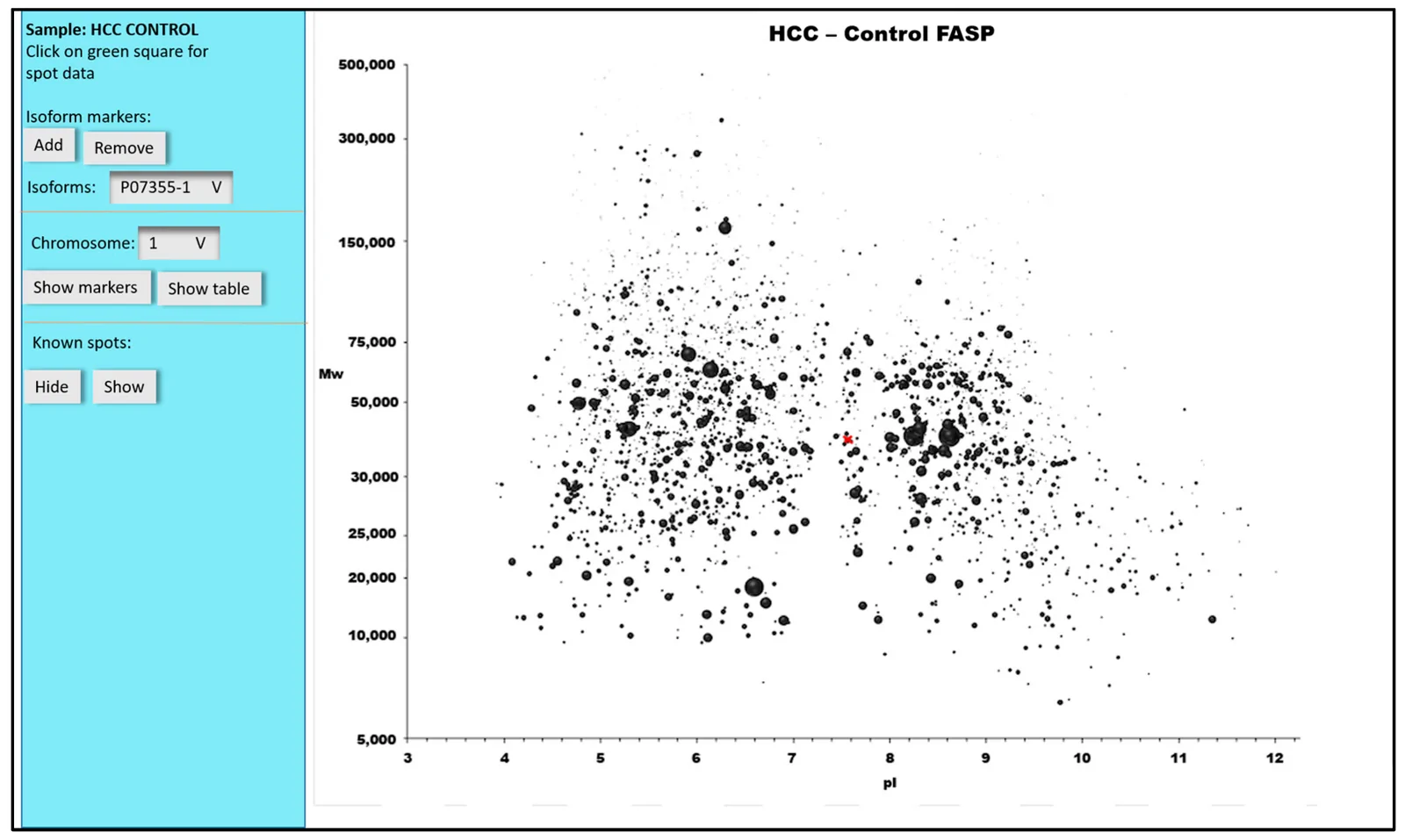
An example of a “Sample Page” on the human proteoform database “2DE-pattern”, where virtual 2DE was used. Information about each spot is presented in the table. A spot of the master form of isoform 1 of ANXA2 (P07355-1) is marked by the red cross.

Virtual 2DE Map: This was used for HCC and HCCCONTROL samples (Figure 3). It constructs a digital map using quantitative FASP analysis of extracts combined with theoretical physical–chemical parameters (pI and MW) of proteins. Virtual 2DE map data provide additional visual representation of the proteome state. Interestingly, the virtual 2DE map of the master protein forms (277 proteins) encoded by human chromosome 18 also resembles a liver or tumor protein map. This provides additional grounds for extrapolating many of the data obtained on chromosome 18 proteins to the entire proteome.

**Figure 4:**
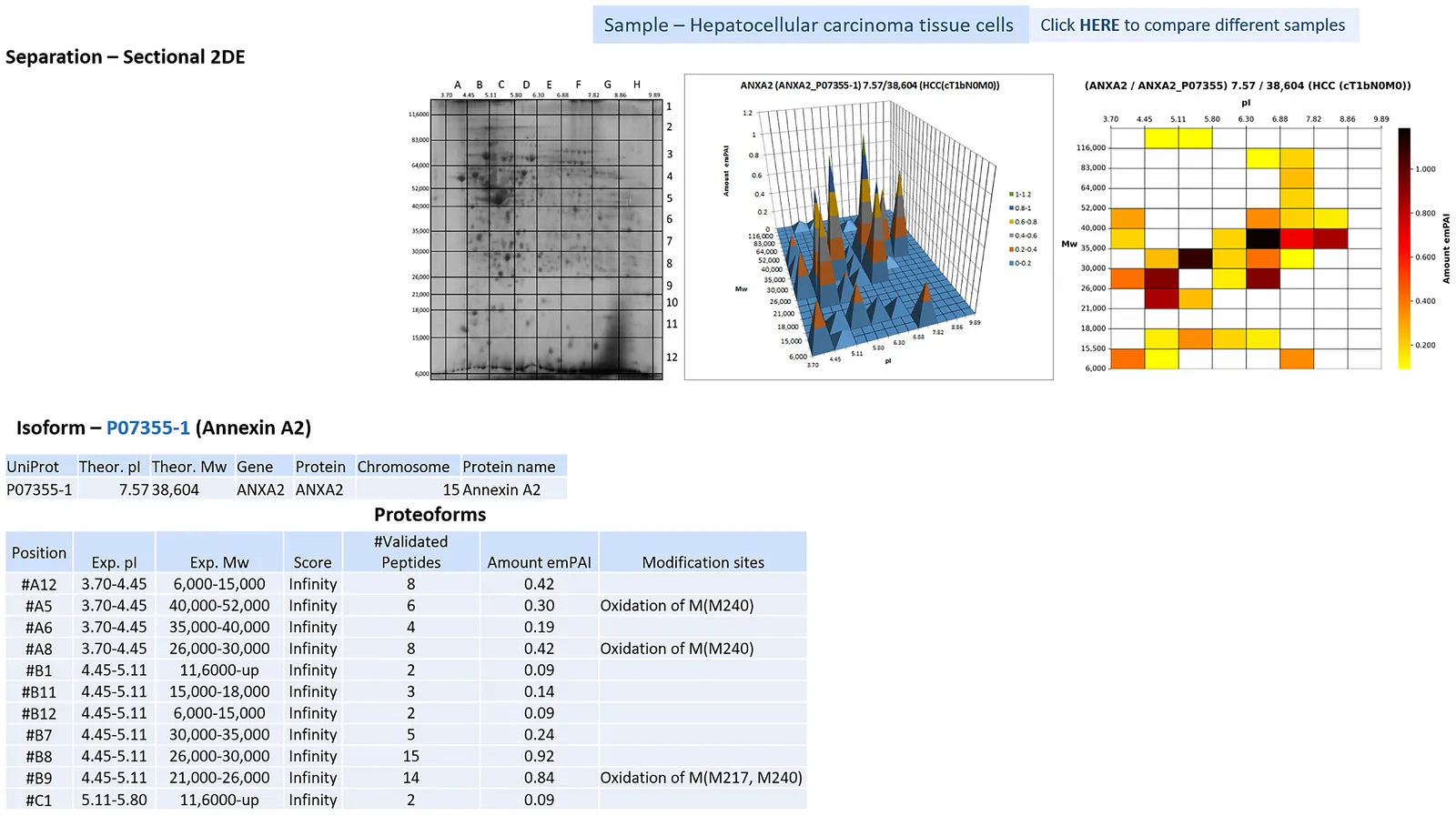
An example of a “Sample Page” with sectional 2DE. A 2DE gel (HCC) divided into sections with determined coordinates is shown on the left. Pyramid-type and heat-map-type distributions of proteoforms among the sections (proteoform pattern) of isoform 1 of ANXA2 (P07355-1) are shown on the right. A table contains basic theoretical information about this isoform (pI/MW, isoform, protein, gene, chromosome). Another table contains experimental data about the proteoforms (pI/MW coordinates of the sections, where the isoform was detected, and MS information—score, number of detected sequences, emPAI, modification sites).

A “Sample Page” with sectional 2DE was also upgraded (Figure 4). To improve perception and analysis, two types of patterns were constructed: volumetric (three-dimensional), in the form of pyramids, where the height of the pyramid of each section corresponded to the level of the proteoform located in this section (the emPAI parameter); and in the form of heat maps, where the color corresponded to the level of the proteoform. A “Sample Page” with semi-virtual 2DE looks the same as it did when the database was launched in 2020.

All the different approaches have advantages and disadvantages. The data obtained by classical experimental 2DE have high resolution but are limited in terms of the number of proteoforms, as they were detected only in the stained spots. Virtual 2DE allows the user to get unbiased images of the proteomes, which are convenient to compare with each other. But they contain only the master forms of proteins and are missing information about other proteoforms. While sectional 2DE suffers from low resolution and overlapping proteoform signals, it maps the entire gel to detect invisible, unstained proteoforms. The pattern obtained by semi-virtual 2DE has high enough resolution in the pH direction but is missing information about the real MW of the proteoforms. Integrating information from these four approaches, a user can reveal a comprehensive, “ideal” proteoform profile. The “Sample page” contains direct links to external databases for deeper analysis. Users can access UniProt to view known post-translational modifications (PTMs) for the isoform. Alternatively, they can use the Proteoform Atlas to check if specific proteoforms have been previously detected via top-down mass spectrometry.

## 4. Conclusions

This paper describes an upgraded version of the database “2DE-pattern”, which was developed for storing and analyzing information about patterns or profiles of human proteoforms generated according to 2DE principles [29]. The database can be freely used at http://2de-pattern.pnpi.nrcki.ru. A central theme here is the necessity of 2DE in an era dominated by mass spectrometry [23,40]. Critics often argue that high-resolution mass spectrometers can now allow identifying thousands of proteins in a single run, seemingly rendering the “visual” approach of the 2DE-pattern database obsolete [41]. However, this argument overlooks the difference between identification and characterization [42]. Our approach allows generating an “integral image” of proteoform distribution according to their physicochemical parameters. This allows performing a direct comparison between normal and cancer samples to find changes that are involved in malignant transformation. This comparison can enable discovering the presence of specific proteoforms suitable for use as biomarkers or as targets for therapy. A panel of such proteins for hepatocarcinoma was revealed recently [28]. The complete characterization of these proteoforms is a more difficult problem to solve. It seems that top-down MS can provide a solution to this task. Indeed, there has been major progress in this area, as the Proteoform Atlas now has data about 66496 proteoforms detected in human cells (http://human-proteoform-atlas.org/ accessed on 22 May 2026). What is important is that the data from the Proteoform Atlas and integrative top-down proteomics (2DE-pattern) can be complementary to each other [25,28]. The primary challenge lies in unifying these experimental results. Implementing data standardization and unification protocols would provide significant benefits. Specifically, integrating key metadata—such as the average molecular weight (MW) and calculated isoelectric point (pI) for each entry—into the Proteoform Atlas would enable direct cross-database comparisons, particularly with the “2DE-pattern” database.

The future of proteoform-based proteomics will depend on the systematic integration of proteoform mapping, functional characterization, and predictive modeling. This vision requires progress in several areas that will enhance the translational value of proteoform research [43,44,45]. Maintaining and replenishing the proteoform databases should surely be a basis of this process [46].

## Data Availability

The data that support the findings of this study are openly available at http://2de-pattern.pnpi.nrcki.ru/index.html (accessed on 19 June 2026) and https://proteomecentral.proteomexchange.org/ui?pxid=PXD010142 (accessed on 19 June 2026).

## Abbreviations

The following abbreviations are used in this manuscript:

2DE	two-dimensional electrophoresis
ESI LC-MS/MS	liquid chromatography–electrospray ionization–tandem mass spectrometry
DTT	dithiothreitol
MS	mass spectrometry
PTM	post-translational modification
emPAI	exponential modified form of protein abundance index
SIB	Swiss Institute of Bioinformatics
